# Lectures on the Theory and Practice of Physic

**Published:** 1842-12-24

**Authors:** 


*Lectures on the Theory and Practice of Physic.* By William Stokes, M. D., Lecturer at the Medical School, Park street, Dublin; Physician to the Meath Hospital, etc., etc. And by John Bell, M. D., Lecturer on Materia Medica and Therapeutics; Fellow of the College of Physicians of Philadelphia, etc., etc. Second edition. In two volumes. Philadelphia: Ed. Barrington & Geo. D. Haswell.

This is the third American edition of Dr. Stokes' work on the Practice of Medicine. His other work, that on the diseases of the lungs, was published in a separate volume in Ireland, and reprinted in this country, and is now, in great part, incorporated with the present edition, but *remodeled* by Dr. Bell.

The editor gives the following account of the preparation of the present edition.

"It was not without some misgivings that I undertook, two years ago, to edit the Lectures of Dr. Stokes,—fearing that my additions might not enhance the value of this gentleman's labours, and knowing that adequate scope was not allowed me for separate efforts. The rapid sale of a large edition of our joint work has, however, reassured me on these points, while it has imparted fresh desire to contribute a fuller share on my part than was required of me on the former occasion. In order to preserve a harmony, in one respect at least, between the British and American portions of the present edition, I have continued the lecture style throughout.

" By the large additions which it has received, the work is now made to assume the character of a System of Medicine more than heretofore; at least, the circle of the most important and violent diseases, those which constitute the chief outlet to human life, is in a great measure complete. It consists at present of the following classes of diseases:—I. *The Digestive System;* II. *The Biliary Apparatus;* III. *The Spleen and Pancreas;* IV. *The Urinary Apparatus;* V. *The Respiratory Apparatus;* VI. *The Heart;* VII. *The Nervous System;* VIII. *Fevers.* Of these classes, the third, fourth, fifth, and sixth, are contributed for the first time, and constitute large and material improvements in the present edition. To the first class, or the Diseases of the Digestive System, my additions, both on the score of number and detail, have been considerable."

"In preparing the lectures on the Diseases of the Respiratory System I have incorporated much valuable matter derived from the work of Dr. Stokes on the *Diagnosis and Treatment of Diseases of the Chest*, besides that which appears in a lecture form, as directly from him, but, also, taken from the work just mentioned. On this last point, an explanation, if not apology, is due to Dr. Stokes,—not for any change of language or train of argument; for the text, with the exception of a few words, has not been altered, but for my arranging in *quasi* lectures some of the matter which he published in a volume, without his having previously presented it in this shape. The only difference, however, between the originally printed matter and that which I have introduced formally as from Dr. Stokes, is in its receiving the division and headings of lectures. Perhaps less objection will be found with this innovation, as there was not even a division into chapters in the work on Diagnosis and Treatment of the Diseases of the Chest.

The class of Fevers has not been treated so much *in extenso* as precedent would have warranted; but by withholding common place literature, and the mystical disquisitions of the past age, without, however, yielding too much to the arithmetical affectation of the present, and by steadily bearing in mind the wants and expectations of the American practitioner for information respecting the fevers of the United States and analogous climates, rather than those of European hospitals, camps, and jails, less disappointment will, it is hoped, be felt at my abbreviations on this head. I have curtailed to some extent my former lectures on Congestive Fever, but have still retained those distinctive features which imparted to them that interest in the minds of the physicians of the South and West, which I was sanguine enough to anticipate when I first took up the subject. Let me, in conclusion, exhort them to send back to us in the city, in return for our issues, full and carefully prepared histories of their fevers; for a complete elucidation of the nature and treatment of which they must not look to the hospital statistics nor collegiate teaching of Europe, without the aid which it is in their power so amply to supply."

The nature of this work does not admit of an analysis, nor indeed is it necessary to enter fully into the merit of a work which in one form or another has been so long before the public, and has been received with so much favour.

The recent additions include almost every disease usually treated of, in a work on the Practice of Medicine. They contain much instructive matter, and will be profitably read by the Student. Doctor Bell's experience, as an American practitioner, has shown him what subjects require to be treated of more at large than is necessary in a work published in Great Britain, where the climate is much more temperate; and he has availed himself of this experience to make such additions as the difference in the forms of the diseases of the two countries require, to make the work more complete for American practitioners. The least favourable specimens are some of his remarks upon continued fever, which are not a little obscure, especially those which relate to the specific character of these diseases.

The course adopted by Dr. Bell may expose him to some remark; for our own part we do not admit the propriety of thus remodeling the works of an author,—at least of one still living. There may be circumstances which may render it proper with the writings of one not a cotemporary. In expressing our objections we give merely our individual opinion, being aware that Dr. Bell has been guided by the views which he believes to be right, although not sanctioned by general custom, and, as we think, for obvious reasons objectionable.

